# An Exploration of Shared Risk Factors for Coronary Artery Disease and Cancer from 109 Traits: The Evidence from Two-Sample Mendelian Randomization Studies

**DOI:** 10.31083/j.rcm2507245

**Published:** 2024-07-03

**Authors:** Rong Xu, Rumeng Chen, Shuling Xu, Yining Ding, Tingjin Zheng, Chaoqun Ouyang, Xiaoming Ding, Linlin Chen, Wenzhou Zhang, Chenjin Ge, Sen Li

**Affiliations:** ^1^Department of Pharmacy, Quanzhou Medical College, 362011 Quanzhou, Fujian, China; ^2^School of Life Sciences, Beijing University of Chinese Medicine, 102488 Beijing, China; ^3^Department of Clinical Laboratory, Quanzhou First Hospital Affiliated to Fujian Medical University, 362000 Quanzhou, Fujian, China; ^4^Department of Basic Medicine, Quanzhou Medical College, 362011 Quanzhou, Fujian, China; ^5^Department of Medical Imaging, Shanghai Municipal Hospital of Traditional Chinese Medicine, Shanghai University of Traditional Chinese Medicine, 200071 Shanghai, China

**Keywords:** coronary artery disease, cancer, shared risk factor, Mendelian randomization, UK Biobank

## Abstract

**Background:**

Although observational studies have reported several common biomarkers related to coronary artery disease (CAD) and cancer, there is a shortage of traditional epidemiological data to establish causative linkages. Thus, we conducted a comprehensive two-sample Mendelian randomization (MR) analysis to systematically investigate the causal associations of 109 traits with both CAD and cancer to identify their shared risk and protective factors.

**Methods:**

The genetic association datasets pertaining to exposure and outcomes were reviewed using the most recent and public genome-wide association studies (GWAS). Inverse variance weighting (IVW), weighted median (WM), and MR-Egger strategies were implemented for the MR analyses. The heterogeneity and pleiotropy were measured utilizing leave-one-out sensitivity testing, MR-PRESSO outlier detection, and Cochran’s Q test.

**Results:**

The IVW analyses revealed that genetic-predicted mean sphered cell volume (MSCV) is a protective factor for CAD, and weight is a risk factor. MSCV and weight also show similar effects on cancer. Furthermore, our study also identified a set of risk and protective factors unique to CAD and cancer, such as telomere length.

**Conclusions:**

Our Mendelian randomization study sheds light on shared and unique risk and protective factors for CAD and cancer, offering valuable insights that could guide future research and the development of personalized strategies for preventing and treating these two significant health issues.

## 1. Introduction

Both coronary artery disease (CAD) and cancer are significant health issues [1, 2], and account for two-thirds of all illness-related deaths worldwide, and they commonly overlap [3]. Sixty-six percent of cancer patients developed an acute coronary syndrome [4]. A Swedish cohort study discovered that the majority of cancers were linked to an increased risk of coronary heart disease within the initial 6 months following their diagnosis [5]. Furthermore, a systematic evaluation and meta-analysis of a cohort study revealed that patients with coronary artery disease had a significantly higher risk of developing cancer compared to those without coronary artery disease [6]. CAD and cancer frequently manifest in overlapping patient populations due to shared biological mechanisms and common risk factors [7].

Despite the existence of numerous studies investigating the factors influencing both CAD and cancer, the conclusions drawn from these studies are inconclusive and inconsistent. For example, testosterone has been demonstrated to raise noncalcified plaque volume in heart arteries [8, 9]. Another set of epidemiological and observational studies reached the opposite conclusion: low testosterone levels correspond with atherosclerosis (AS) [10]. Similarly, studies have shown that testosterone, by binding to androgen receptors, inhibits the risk of breast cancer [11]. On the other hand, Davis [12] identified no correlation between testosterone and female breast cancer or other aggressive tumors. Hence, additional research is warranted to further investigate the shared underlying factors contributing to both CAD and cancer.

Because of unknown residual confounding, observational research may be restricted in the ability to establish causal relationships between risk factors and disorders [13]. Thus, we performed Mendelian randomization (MR) analyses to identify shared risk factors between CAD and cancer and explore their causal relationships. MR is a statistical method to assess the association between putative risk factors or exposures and medical results [14, 15, 16]. It can mitigate the obscuring effects of reverse causality and residual confounding using instrumental variables (IVs) that mimic randomization of individual exposure, hence safeguarding the strength of causal chains. This study adds to our knowledge of the causal associations between as many as 109 potential risk factors and CAD/cancer, offering opportunities for individualized targeted preventive and treatment therapies.

## 2. Methods

### 2.1 Study Design 

MR is an epidemiological method that aims to determine the presence of a causal relationship between exposure and outcome by utilizing IVs. The three steps of MR are as follows: (1) Selection of exposure and outcome data from the available genome-wide association study (GWAS), followed by the selection of single nucleotide polymorphism (SNPs) as IVs. (2) Utilization of IVs to assess the presence of causal relationships between exposure and outcome using various statistical methods of MR analysis. (3) Conducting a sensitivity analysis to verify the reliability of the MR results.

### 2.2 Data Sources

Utilizing a published trait selection method (**Supplementary Fig. 1**) described by Walker *et al*. [17], we utilized GWAS summary statistics that were minimally adjusted for the specific traits. These statistics were derived from the largest population of individuals with European or mixed ancestry, comprising both genders, in the Integrative Epidemiology Unit (IEU) OpenGWAS database. Omic variables related to gene expression, protein level and metabolomics were excluded from our analyses.

The GWAS analyses for the investigated traits mainly originated from the UK Biobank (UKBB), while the GWAS studies examining cancer susceptibility and liability to CAD were obtained from FinnGen and the CARDIoGRAM consortium [18], respectively (**Supplementary Table 1**). More detailed information can be found on the websites of IEU OpenGWAS (https://gwas.mrcieu.ac.uk/), the FinnGen (https://www.finngen.fi/), and the CARDIoGRAM consortium (http://www.cardiogramplusc4d.org/).

### 2.3 Selection of IVs

The three essential assumptions for IVs selection are outlined as follows: (1) IVs should exhibit an association with the exposure; (2) IVs should not be associated with any confounding factors; and (3) IVs should solely influence the outcome through the exposure. To ensure compliance with these assumptions, specific implementation criteria were established as described in previous publications [19, 20, 21]. The inclusion criteria mandated a robust genetic association between the IVs and the exposure of interest, as demonstrated by a *p* < 5 × ${\mathrm{10}}^{-8}$. Independent IVs with minimal levels of linkage disequilibrium, indicated by an $R^{2}$ value below 0.001, were detected through the application of the clumping method within a genomic interval of 10 megabases. Consistent with previous research findings, only IVs with minor allele frequencies exceeding 0.01 were considered for analysis. F-statistics were computed as indicators of IV strength, where values exceeding ten indicate minimal vulnerability to weak instrument bias [22].

### 2.4 MR Analysis

The primary approach utilized in this study among the three MR methods employed was the inverse-variance weighted (IVW) method. To address situations where the IVW result lacks sufficient accuracy, the weighted median (WM) method was employed [23]. Additionally, the IVW method assumes that its intercept must pass through zero [24], which may overlook cases where the intercept deviates from zero. To account for this limitation, the MR-Egger method was utilized as a supplementary approach [25]. We utilized Steiger filtering analysis to identify SNPs exhibiting an incorrect causal direction [26]. Furthermore, we conducted the MR Steiger directionality test to explore the direction of the effect in the exposure-outcome association.

### 2.5 Sensitivity Analysis

In order to assess the possibility of horizontal pleiotropy, we performed a test on the intercept using the MR-Egger method. Additionally, the identification of SNPs exhibiting horizontal pleiotropy was conducted using PhenoScanner v2 [27, 28]. To accommodate for potential outliers, we integrated pleiotropy-corrected data obtained from MR-PRESSO. The Cochrane Q value was computed in order to evaluate the presence of heterogeneity. A leave-one-out sensitivity analysis was conducted to investigate the impact of individual IVs on causal relationships and verify the reliability of the findings. In the MR analyses, the assessment of causal effects involved odds ratios (ORs), along with their respective 95% confidence intervals (95% CIs), given that the outcome variable was dichotomous. To account for the issue of multiple comparisons, a false discovery rate (FDR) threshold of 5% was utilized. The TwoSampleMR package in R 4.2.2 was utilized to perform all MR analyses.

## 3. Results

### 3.1 Assessment of the IVs

MR analysis was employed to investigate the relationships between 109 traits and both CAD and cancer. The IVs corresponding to the potential factors exhibited F-statistics ranging from 29.73 to 8023.61. These F-statistics signify robust instrument strength, as outlined in **Supplementary Table 2**.

### 3.2 Results of the MR Analysis

**Fig. 1.**
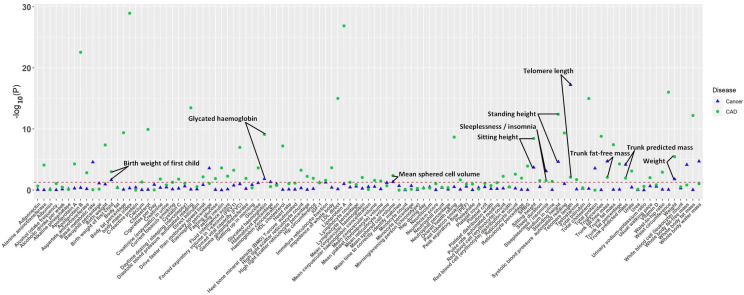
**The *p*-value distribution of associations between 109 factors and both CAD and cancer in the Mendelian randomization analysis.** The read dashed line represents the suggestive significance threshold, set at *p* = 0.05. CAD, coronary artery disease.

**Fig. 2.**
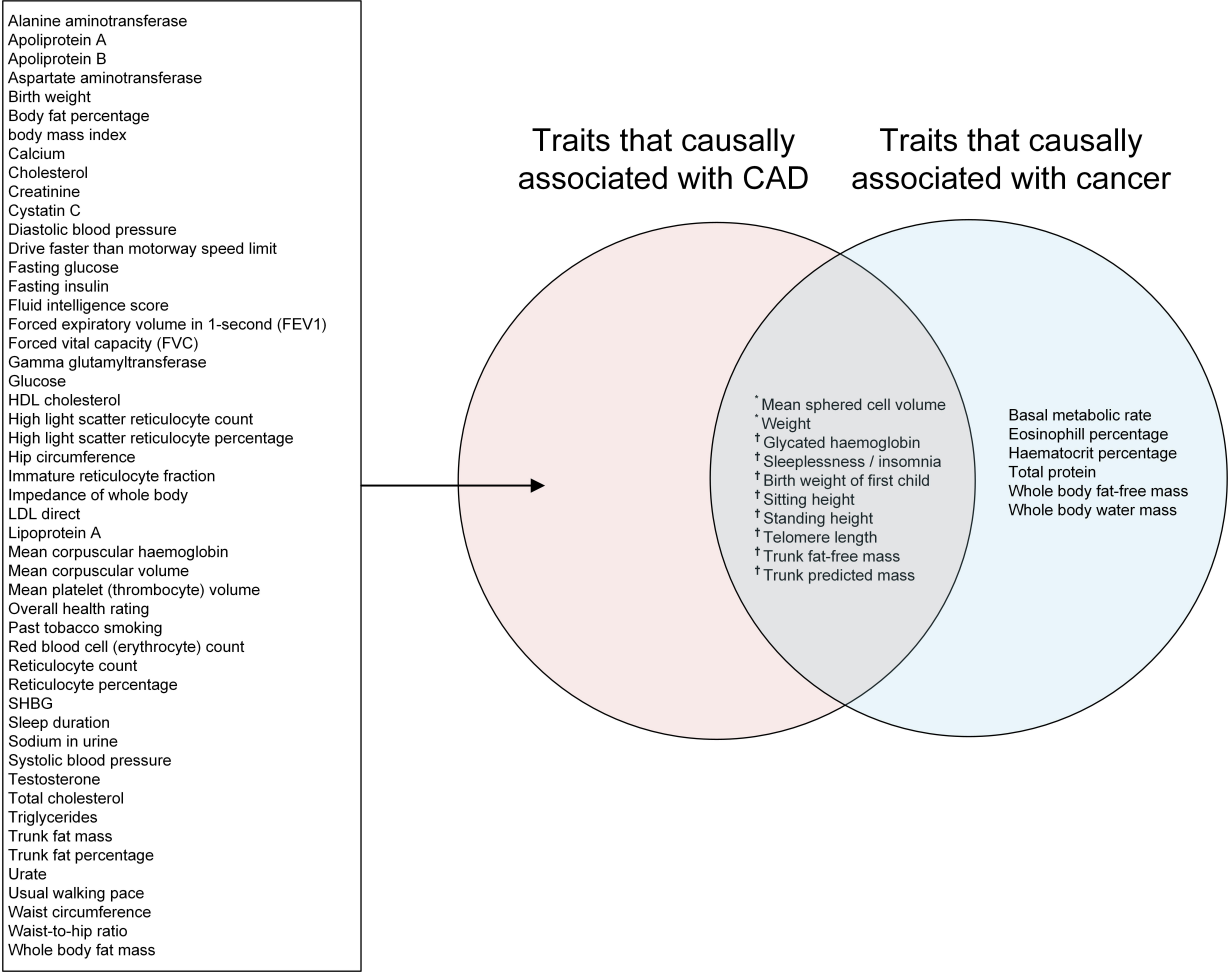
**Venn diagram showing traits causally associated with CAD, cancer, or both.** * indicates traits associated with both CAD and cancer, with the direction of the associations being the same. ^†^ indicates traits associated with both CAD and cancer, but with different directions of association with these two diseases. CAD, coronary artery disease; HDL, high-density lipoprotein; LDL, low-density lipoprotein; SHBG, sex hormone binding globulin.

The IVW analysis revealed suggestive causal associations between 60 factors and CAD (*p* < 0.05), as well as between 16 factors and cancer (*p* < 0.05). Furthermore, 10 factors exhibited suggestive causal associations with both CAD and cancer (*p* < 0.05) (Figs. 1,2).

**Fig. 3.**
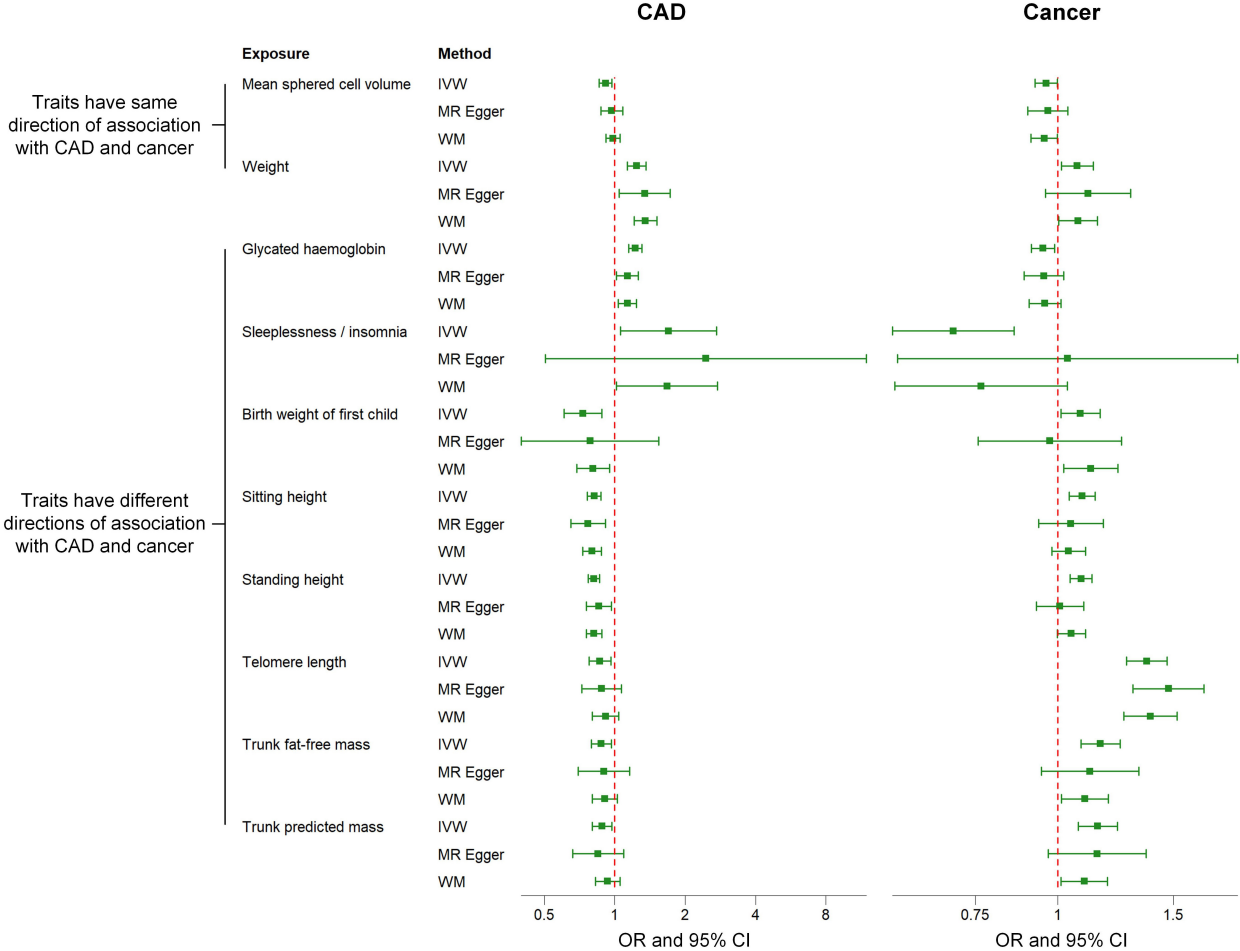
**Comparison of associations between genetically predicted 10 factors on CAD and cancer examined by three MR methods. **IVW methods indicate that the directions of the associations between two factors (i.e., mean sphered cell volume and weight) and CAD are the same as those of the associations between these two factors and cancer. The directions of the associations between the other eight factors (i.e., telomere length, sleeplessness or insomnia, sitting height, trunk fat-free mass, trunk predicted mass, birth weight of the first child, standing height, glycated hemoglobin) and CAD are different from those of the associations between these eight factors and cancer. MR, Mendelian randomization; IVW, inverse-variance weighted; WM, weighted median; OR, odds ratio; CI, confidence interval; CAD, coronary artery disease.

However, out of these 10 factors, only mean sphered cell volume (MSCV) and weight exhibited the same association direction with CAD and cancer, while the direction of associations between the other eight traits and CAD was different from that of cancer (Fig. 3).

**Fig. 4.**
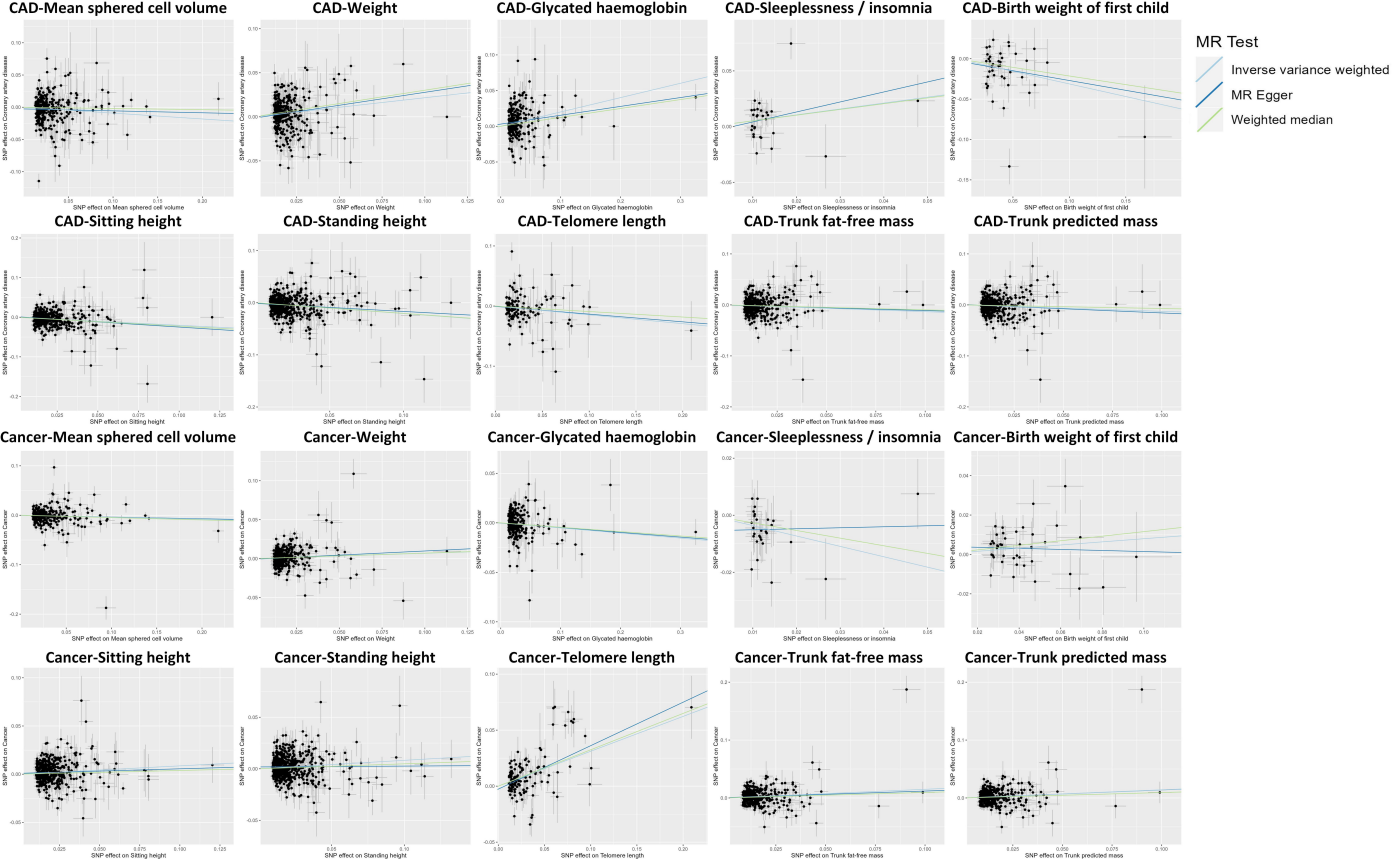
**Scatter plot showing the causal effects of 10 factors on CAD and cancer.** IVW results in the scatter plots indicate that the directions of the associations between two factors (i.e., mean sphered cell volume and weight) and CAD are the same as those of the associations between these two factors and cancer. The directions of the associations between the other eight factors (i.e., telomere length, sleeplessness or insomnia, sitting height, trunk fat-free mass, trunk predicted mass, birth weight of the first child, standing height, glycated hemoglobin) and CAD are different from those of the associations between these eight factors and cancer. SNP, single nucleotide polymorphism; CAD, coronary artery disease; MR, Mendelian randomization; IVW, inverse-variance weighted.

The remaining eight factors that causally associated with both CAD and cancer, such as telomere length (TL) and glycated hemoglobin (HbA1c), exhibited opposite association directions with CAD and cancer, as outlined in Fig. 3. Specifically, a positive causal relationship was observed between TL and cancer, while a negative causal relationship was identified with CAD. Conversely, HbA1c exhibited a suggestive negative causal relationship with cancer and a suggestive positive causal relationship with CAD. The relationships between potential risk factors and both CAD and cancer were further examined using the MR-Egger and WM approaches, and consistent results for most traits were observed (Fig. 3). Furthermore, similar results were obtained when using instrumental SNPs after Steiger filtering (**Supplementary Table 3**). The results we observed also followed the expected direction as revealed by Steiger directionality test (**Supplementary Table 4**). However, the results indicated that the associations between five factors and CAD/cancer remained significant after multiple comparison correction: sitting height, standing height, telomere length, trunk fat-free mass, and trunk predicted mass. Fig. 4 presents a scatter plot that visually depicts the potential causal relationship between the 10 factors and both CAD and cancer.

### 3.3 Results of the Sensitivity Analysis

**Fig. 5.**
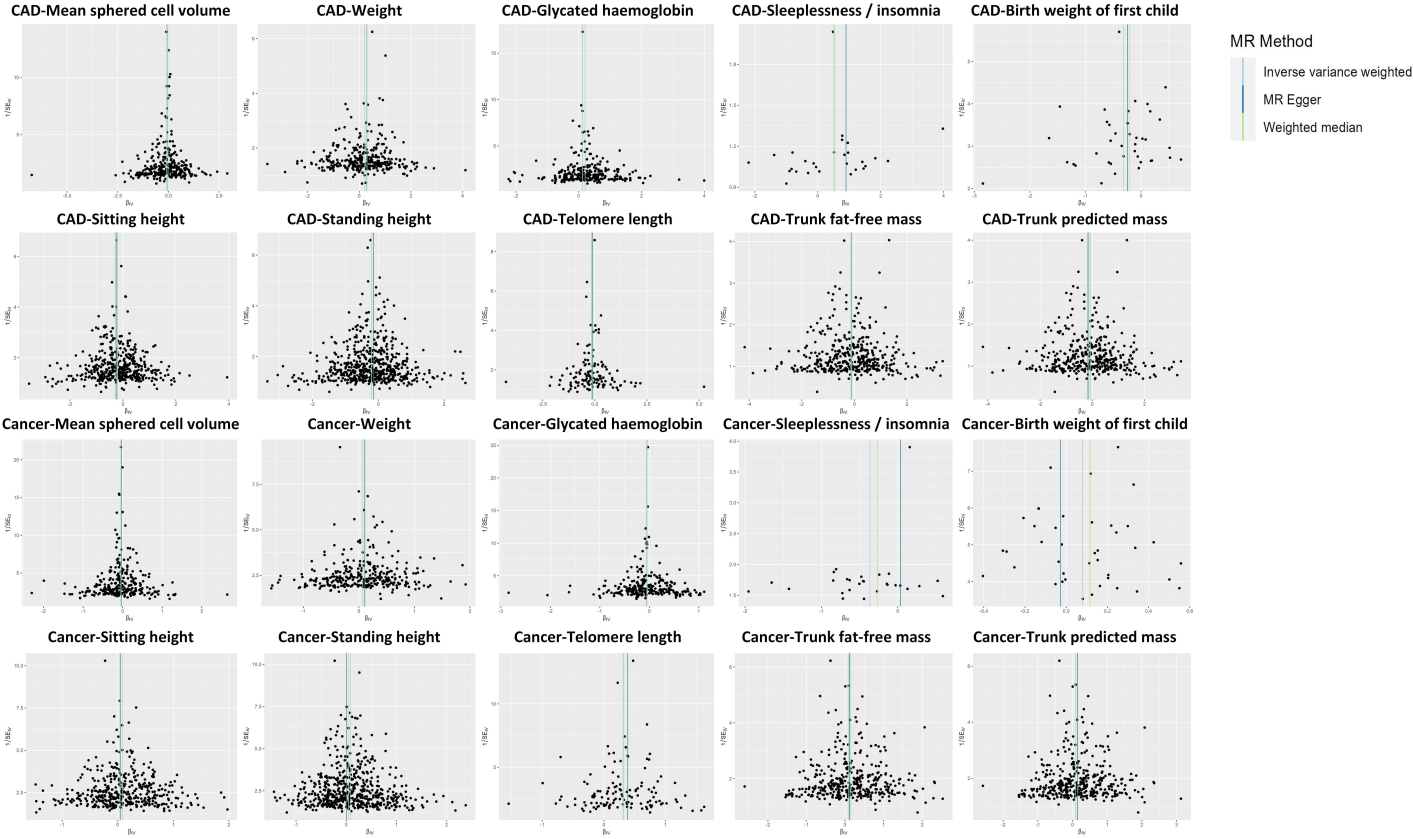
** Funnel plots are employed to depict the heterogeneity of MR estimates concerning the impact of 10 factors (i.e., mean sphered cell volume, weight, telomere length, sleeplessness or insomnia, sitting height, trunk fat-free mass, trunk predicted mass, birth weight of the first child, standing height, and glycated hemoglobin) on the susceptibility to both CAD and cancer.** MR, Mendelian randomization; IV, instrumental variable; SE, standard error; CAD, coronary artery disease.

The potential heterogeneity was investigated (see Fig. 5 and **Supplementary Table 5**), and the analysis of the intercept term using the MR-Egger method did not show substantial indications of horizontal pleiotropy in the majority of the assessments (see **Supplementary Table 6**). Although MR-PRESSO identified outlier IVs, most results were not significantly altered after excluding the outliers (see **Supplementary Table 7**). Furthermore, the majority of our analysis results remained unchanged after excluding SNPs showing associations with CAD or cancer (**Supplementary Table 8**). The results of the leave-one-out method, depicted in **Supplementary Fig. 2**, revealed that the majority of results did not cross the invalid line after removing specific SNP. This observation suggests that there is minimal potential bias present in the study.

## 4. Discussion

In this study, we employed an MR design to investigate potential traits that could influence both CAD and cancer. The results revealed a potential causal relationship between 10 factors and the risk of both CAD and cancer. Among these factors, 2 traits demonstrated consistent association direction for both diseases. Specifically, an increase in MSCV was found to simultaneously decrease the risk of both CAD and cancer, while higher body weight was associated with an increased risk of both diseases. However, the remaining eight factors exhibited opposite effects on CAD and cancer. For instance, TL was found to promote cancer but decrease CAD risk, whereas HbA1c was associated with an increased risk of CAD but a decreased risk of cancer.

### 4.1 Shared Risk and Protective Factors for CAD and Cancer

This study provides insights into a potential inverse causal relationship between MSCV and both CAD and cancer. However, a machine learning study contradicts our findings by reporting MSCV as a predictor of CAD [29]. This discrepancy is likely due to the machine learning data predominantly originating from cohort studies, raising concerns about confounding factors and reverse causality. Nonetheless, a prospective study based on the UK Biobank supports our findings by revealing an association between MSCV and a reduced risk of prostate cancer [30]. The potential mechanism underlying the impact of MSCV on CAD and cancer may involve preserving endothelial cell (EC) functional stability. A cross-sectional study conducted with elderly Japanese adults found a negative association between reticulocyte (RET) levels and AS [31]. EC damage subsequently induces inflammation, mononuclear cell infiltration, and activation of vascular smooth muscle cells, leading to the development of AS [32, 33, 34]. Elevated levels of RET may contribute to preserving EC stability [31]. Furthermore, it has been observed that morphologically aberrant tumor vasculature facilitates the intravasation of tumor cells during metastasis. Previously, tumor blood vessels and tumor endothelial cells (TECs) were presumed to be identical to their normal EC [35]. Based on this, we hypothesize that increased RET levels may stabilize the function of TECs and inhibit the intravasation of tumor cells during metastasis. MSCV is frequently used as an additional parameter for assessing RET counts [36]. Hence, it is plausible to speculate that elevated MSCV levels may confer protective effects against both CAD and cancer by preserving stable EC function.

The present study has established a potential positive causal relationship between weight and both CAD and cancer, consistent with previous research findings. Previous studies have independently identified obesity as a risk factor for CAD [37], with obesity contributing to 11.9% of cancer cases in men and 13.1% in women [38]. The International Agency for Research on Cancer working group has also reported that overweight or obesity increases the risk of at least 13 types of cancer [39]. Additionally, overweight or obesity, particularly when accompanied by frequent weight fluctuations, significantly amplifies the risk of coronary events [40]. Macroscopic simulation models have further demonstrated that weight loss can prevent numerous future cancer cases over a 30-year period from 2020 to 2050 [41]. However, the precise mechanisms through which obesity contributes to CAD and cancer in patients remain incompletely understood. It is hypothesized that this association may be attributed to endocrine dysfunction of adipose tissue resulting from weight gain. In obese individuals, excessive accumulation of adipose tissue leads to endocrine dysfunction, which in turn promotes the proliferation of pro-inflammatory adipokines. These adipokines subsequently lead to endothelial dysfunction and inflammation, ultimately contributing to the development of atherosclerosis and cancer [37, 42].

### 4.2 Other Traits Causally Associated with CAD and Cancer

Our study has revealed a potential positive causal relationship between TL and cancer, as well as a potential negative causal relationship between TL and CAD. Shorter TL may signal atherosclerotic thrombosis, and genetic research in humans suggest an inverse causal relationship between leukocyte telomere length and CAD [43, 44]. Telomere shortening caused by aging or an unhealthy lifestyle is accompanied by a decrease in telomerase activity, which contributes to increased cellular oxidative stress, elevated levels of inflammatory processes arbitrators, and other related complications [45]. In terms of cancer, maintaining telomeres matters for cancer development [46]. Cancer cells achieve replicative immortality by triggering telomere maintenance mechanisms, including telomerase and alternative lengthening of telomeres routes [47]. Therefore, these observational studies also support our causal connection between TL and both cancer and CAD.

Our study has found a potential positive causal relationship between HbA1c and CAD. Elevated HbA1c levels alone constitute a potential cause of CAD [48]. HbA1c has an undesirable effect on the coronary arteries, resulting both in stenoses (a 0.02 mm rise in coronary intima-media thickness per 1% increase in HbA1c) and a higher rate of vascular lesions [49, 50]. Furthermore, a MR study has provided evidence of a positive association between genetically predicted HbA1c and CAD [51], which is consistent with the findings of our study. The underlying mechanism involves the activation of inflammatory pathways mediated by advanced glycation end products (AGEs) due to increased HbA1c levels. HbA1c serves as an indicator of chronic hyperglycemia, as it undergoes non-enzymatic glycation of hemoglobin [52]. Chronic hyperglycemia leads to the production of reactive oxygen species (ROS) and the accumulation of AGEs. Additionally, it results in the expression of the receptor for advanced glycation end products (RAGE) and RAGE ligands [53, 54]. The accumulation of ROS directly damages blood vessels and triggers downstream cellular pathways mediated by AGEs. This cascade leads to the production of several inflammatory factors, including vascular cell adhesion molecule-1. Ultimately, these processes contribute to arterial stiffness, vascular calcification, and the accumulation of plaque [54].

Various research studying the relationship between HbA1c and cancer have shown contradictory results. A Korean cohort study consisting of 7822 participants without a history of cancer or diabetes at baseline found that higher levels of circulating HbA1c were associated with an increased risk of total cancer in the Korean population [52]. However, HbA1c and blood glucose levels had little influence on the survival of people who died within five years after being diagnosed with cancer [55]. Furthermore, a UK cohort study with 378,253 participants did not establish any independent positive association between HbA1c and the risk of other cancers, except for pancreatic cancer [56]. A multicenter prospective cohort study conducted in China involving 193,846 participants found that 2-hour postprandial blood glucose, but not fasting glucose or HbA1c, was associated with overall cancer risk [57]. Our study has identified a potential negative causal relationship between HbA1c and cancer, which contradicts previous studies. In studies with multiple outcomes, such as CAD and cancer, competing risk events may occur when the potential competition between these endpoints is ignored [58]. This may explain our observation that HbA1c is a risk factor for CAD but has a protective effect against cancer: individuals with high levels of HbA1c may develop CAD and die from it before developing cancer, thereby decreasing their cancer risk. The analyses of other traits (e.g., height, trunk mass, and insomnia) showing opposite associations with CAD and cancer may also be affected by the potential competitive risks of the outcomes. Thus, future research is warranted to further investigate these associations.

### 4.3 Strengths and Weaknesses

Our study has several notable strengths. First, the utilization of MR studies provides distinct advantages in comparison to observational studies. By leveraging genetic variation as an IV, this study successfully addresses the impact of confounding factors and reverse causality. This methodology offers improved efficiency in terms of time, labor, cost, and ease of implementation when contrasted with randomized controlled trials. Second, we conducted an innovative examination of the causal association between all feasible factors and the occurrence of both CAD and cancer. Furthermore, we performed multiple sensitivity analyses to bolster the reliability and validity of our findings.

Our study also has some limitations. First, the GWAS database we utilized predominantly consists of individuals with European ancestry, which may restrict the applicability of our findings to populations of diverse ancestries. Second, our analyses can infer causality only based on linear associations, and we cannot definitively conclude that the relationship between these potential factors and the occurrence of both CAD and cancer strictly adheres to linear causality. Third, we utilized IVs across multiple gene regions (polygenic MR). Colocalization analyses can be applied to investigate whether two traits are influenced by shared or distinct causal variants when focusing on a specific locus. Finally, as previously noted, the results could have been influenced by selection bias, as the GWAS may not have included individuals who passed away due to exposure or competing risk of outcomes.

## 5. Conclusions

In our study, we have identified MSCV as a potential protective factor against both CAD and cancer. Conversely, weight has been recognized as a potential risk factor for the development of CAD and cancer. Moreover, our research also recognized a distinct set of factors that pose risks or offer protection specific to CAD and cancer, including factors like telomere length. This contributes to a better understanding of the similar and different biological foundations of these two conditions, offering valuable insights that could guide future research and the development of personalized strategies for preventing and treating these significant health issues.

## Data Availability

The present study examined the exposure data obtained from the UK Biobank (UKBB) and the outcome data obtained from the FinnGen and CARDIoGRAM consortium. The exposure dataset is publicly available and can be accessed on the IEU Open GWAS Project website (https://gwas.mrcieu.ac.uk/). The dataset specifically related to cancer can be found on the website (https://www.finngen.fi/), while the dataset pertaining to CAD is also publicly accessible and can be found on the website (http://www.cardiogramplusc4d.org/).

## Abbreviations

CAD, coronary artery disease; AS, atherosclerosis; MR, Mendelian randomization; IVs, instrumental variables; GWAS, genome-wide association study; SNPs, single nucleotide polymorphism; UKBB, UK Biobank; IVW, inverse-variance weighted; WM, weighted median; ORs, odds ratios; CIs, confidence intervals; FDR, false discovery rate; MSCV, mean sphered cell volume; TL, telomere length; HbA1c, glycated hemoglobin; HR, hazard ratio; EC, endothelial cell; RET, reticulocyte; TECs, tumor endothelial cells; AGEs, advanced glycation end products; ROS, reactive oxygen species; RAGE, receptor for advanced glycation end products.

## Supplementary Material

Supplementary material associated with this article can be found, in the online version, at https://doi.org/10.31083/j.rcm2507245.
