# Supplementary material for: An Exploration of Shared Risk Factors for Coronary Artery Disease and Cancer from 109 Traits: The Evidence from Two-Sample Mendelian Randomization Studies
Source: Rev Cardiovasc Med. 2024 Jul 3;25(7):245. doi: 10.31083/j.rcm2507245 (PMC11317334; doi:10.31083/j.rcm2507245)
Supplement: Supplementary file 1 [file 2153-8174-25-7-245-s1.docx]

**Supplementary Table 1**. Information of GWAS summary datasets used in MR analyses

| Traits | GWAS.ID |
| --- | --- |
| CAD | NA |
| Cancer | C3_CANCER_EXALLC |
| Adiponectin | ieu-a-1 |
| Alanine aminotransferase | ukb-d-30620_irnt |
| Albumin | ukb-d-30600_irnt |
| Alcohol intake frequency. | ukb-a-25 |
| Alcoholic drinks per week | ieu-b-73 |
| Alkaline phosphatase | ukb-d-30610_irnt |
| Apoliprotein A | ukb-d-30630_irnt |
| Apoliprotein B | ukb-d-30640_irnt |
| Aspartate aminotransferase | ukb-d-30650_irnt |
| Basal metabolic rate | ukb-a-268 |
| Basophill percentage | ukb-d-30220_irnt |
| Birth weight | ukb-a-198 |
| Birth weight of first child | ukb-a-318 |
| Body fat | ieu-a-999 |
| Body fat percentage | ukb-a-264 |
| Body mass index | ieu-b-40 |
| Calcium | ukb-d-30680_irnt |
| Cholesterol | ukb-d-30690_irnt |
| Cigarettes per Day | ieu-b-25 |
| C-reactive protein | ukb-d-30710_irnt |
| Creatinine | ukb-d-30700_irnt |
| Creatinine (enzymatic) in urine | ukb-a-333 |
| Current tobacco smoking | ukb-a-16 |
| Cystatin C | ukb-d-30720_irnt |
| Daytime dozing / sleeping (narcolepsy) | ukb-a-15 |
| Diastolic blood pressure automated reading | ukb-a-359 |
| Direct bilirubin | ukb-d-30660_irnt |
| Drive faster than motorway speed limit | ukb-a-8 |
| Eosinophill percentage | ukb-d-30210_irnt |
| Fasting glucose | ieu-b-114 |
| Fasting insulin | ieu-b-116 |
| Fluid intelligence score | ukb-a-196 |
| Forced expiratory volume in 1-second (FEV1) | ukb-a-337 |
| Forced vital capacity (FVC) | ukb-a-336 |
| Gamma glutamyltransferase | ukb-d-30730_irnt |
| Getting up in morning | ukb-a-10 |
| Glucose | ukb-d-30740_irnt |
| Glycated haemoglobin | ukb-d-30750_irnt |
| Haematocrit percentage | ukb-d-30030_irnt |
| Haemoglobin concentration | ukb-d-30020_irnt |
| HDL cholesterol | ukb-d-30760_irnt |
| Heart rate | ieu-a-1056 |
| Heel bone mineral density (BMD) T-score automated | ukb-a-500 |
| High light scatter reticulocyte count | ukb-d-30300_irnt |
| High light scatter reticulocyte percentage | ukb-d-30290_irnt |
| Hip circumference | ukb-a-388 |
| IGF-1 | ukb-d-30770_irnt |
| Immature reticulocyte fraction | ukb-d-30280_irnt |
| Impedance of whole body | ukb-a-269 |
| LDL direct | ukb-d-30780_irnt |
| Lipoprotein A | ukb-d-30790_irnt |
| Lymphocyte count | ukb-d-30120_irnt |
| Lymphocyte percentage | ukb-d-30180_irnt |
| Mean corpuscular haemoglobin | ukb-d-30050_irnt |
| Mean corpuscular haemoglobin concentration | ukb-d-30060_irnt |
| Mean corpuscular volume | ukb-d-30040_irnt |
| Mean platelet (thrombocyte) volume | ukb-d-30100_irnt |
| Mean reticulocyte volume | ukb-d-30260_irnt |
| Mean sphered cell volume | ukb-d-30270_irnt |
| Mean time to correctly identify matches | ukb-a-199 |
| Monocyte count | ukb-d-30130_irnt |
| Monocyte percentage | ukb-d-30190_irnt |
| Morning/evening person (chronotype) | ukb-a-11 |
| Nap during day | ukb-a-12 |
| Neuroticism | ieu-a-1007 |
| Neuroticism score | ukb-a-230 |
| Neutrophill count | ukb-d-30140_irnt |
| Neutrophill percentage | ukb-d-30200_irnt |
| Overall health rating | ukb-a-251 |
| Past tobacco smoking | ukb-a-17 |
| Peak expiratory flow (PEF) | ukb-a-338 |
| Phosphate | ukb-d-30810_irnt |
| Platelet count | ukb-d-30080_irnt |
| Platelet crit | ukb-d-30090_irnt |
| Platelet distribution width | ukb-d-30110_irnt |
| Pulse rate automated reading | ukb-a-3 |
| Red blood cell (erythrocyte) count | ukb-d-30010_irnt |
| Red blood cell (erythrocyte) distribution width | ukb-d-30070_irnt |
| Reticulocyte count | ukb-d-30250_irnt |
| Reticulocyte percentage | ukb-d-30240_irnt |
| SHBG | ukb-d-30830_irnt |
| Sitting height | ukb-a-195 |
| Sleep duration | ukb-a-9 |
| Sleeplessness / insomnia | ukb-a-13 |
| Sodium in urine | ukb-a-335 |
| Standing height | ukb-a-389 |
| Systolic blood pressure automated reading | ukb-a-360 |
| Telomere length | ieu-b-4879 |
| Testosterone | ukb-d-30850_irnt |
| Total bilirubin | ukb-d-30840_irnt |
| Total cholesterol | ieu-a-301 |
| Total protein | ukb-d-30860_irnt |
| Triglycerides | ukb-d-30870_irnt |
| Trunk fat mass | ukb-a-291 |
| Trunk fat percentage | ukb-a-290 |
| Trunk fat-free mass | ukb-a-292 |
| Trunk predicted mass | ukb-a-293 |
| Urate | ukb-d-30880_irnt |
| Urea | ukb-d-30670_irnt |
| Urinary sodium-potassium ratio | ieu-b-72 |
| Usual walking pace | ukb-a-513 |
| Vitamin D | ukb-d-30890_irnt |
| Waist circumference | ukb-a-382 |
| Waist-to-hip ratio | ieu-a-72 |
| Weight | ukb-a-249 |
| White blood cell (leukocyte) count | ukb-d-30000_irnt |
| Whole body fat mass | ukb-a-265 |
| Whole body fat-free mass | ukb-a-266 |
| Whole body water mass | ukb-a-267 |
| Abbreviation: GWAS: genome-wide association study; ID: Identification; MR: Mendelian randomization; ukb: UK Biobank; CAD: Coronary artery disease; HDL: High-density lipoprotein; IGF-1: Insulin-like growth factor 1; LDL: Low-density lipoprotein; SHBG: Sex hormone-binding globulin; NA: Not Applicable. | |

**Supplementary Table 2**. The F-statistics of IVs

| Exposure | Outcome | F-statistic | |
| --- | --- | --- | --- |
|  |  | Min | Max |
| Mean sphered cell volume | CAD | 29.73 | 2824.94 |
| Weight | CAD | 29.73 | 772.58 |
| Glycated haemoglobin | CAD | 29.94 | 8023.61 |
| Sleeplessness / insomnia | CAD | 30.13 | 150.83 |
| Birth weight of first child | CAD | 30.05 | 134.05 |
| Sitting height | CAD | 29.88 | 1146.83 |
| Standing height | CAD | 29.73 | 1335.34 |
| Telomere length | CAD | 29.86 | 1628.82 |
| Trunk fat-free mass | CAD | 29.74 | 630.54 |
| Trunk predicted mass | CAD | 29.75 | 627.97 |
| Mean sphered cell volume | Cancer | 29.73 | 2824.94 |
| Weight | Cancer | 29.73 | 772.58 |
| Glycated haemoglobin | Cancer | 29.94 | 8023.61 |
| Sleeplessness / insomnia | Cancer | 30.13 | 150.83 |
| Birth weight of first child | Cancer | 30.05 | 134.05 |
| Sitting height | Cancer | 29.88 | 1146.83 |
| Standing height | Cancer | 29.73 | 1335.34 |
| Telomere length | Cancer | 29.86 | 1628.82 |
| Trunk fat-free mass | Cancer | 29.74 | 630.54 |
| Trunk predicted mass | Cancer | 29.75 | 627.97 |
| Abbreviation: IVs: instrumental variables; SNP: single nucleotide polymorphism; CAD: Coronary artery disease. | | | |

**Supplementary Table 3.** MR analysis results using instrumental SNPs after Steiger filtering (only results with SNPs identified through Steiger filtering are included)

| Exposure | Outcome | Method | p |
| --- | --- | --- | --- |
| Mean sphered cell volume | CAD | IVW | 2.44E-03 |
| Mean sphered cell volume | CAD | MR Egger | 4.21E-01 |
| Mean sphered cell volume | CAD | WM | 6.06E-01 |
| Weight | CAD | IVW | 1.22E-06 |
| Weight | CAD | MR Egger | 1.36E-02 |
| Weight | CAD | WM | 1.02E-07 |
| Glycated haemoglobin | CAD | IVW | 6.75E-10 |
| Glycated haemoglobin | CAD | MR Egger | 1.48E-02 |
| Glycated haemoglobin | CAD | WM | 4.52E-03 |
| Sleeplessness / insomnia | CAD | IVW | 7.20E-02 |
| Sleeplessness / insomnia | CAD | MR Egger | 7.85E-01 |
| Sleeplessness / insomnia | CAD | WM | 4.85E-02 |
| Birth weight of first child | CAD | IVW | 9.79E-04 |
| Birth weight of first child | CAD | MR Egger | 6.17E-01 |
| Birth weight of first child | CAD | WM | 9.83E-03 |
| Sitting height | CAD | IVW | 1.00E-09 |
| Sitting height | CAD | MR Egger | 3.87E-03 |
| Sitting height | CAD | WM | 1.45E-06 |
| Telomere length | CAD | IVW | 1.94E-03 |
| Telomere length | CAD | MR Egger | 1.08E-01 |
| Telomere length | CAD | WM | 1.94E-01 |
| Trunk fat-free mass | CAD | IVW | 1.29E-02 |
| Trunk fat-free mass | CAD | MR Egger | 4.27E-01 |
| Trunk fat-free mass | CAD | WM | 1.40E-01 |
| Trunk predicted mass | CAD | IVW | 1.83E-02 |
| Trunk predicted mass | CAD | MR Egger | 2.11E-01 |
| Trunk predicted mass | CAD | WM | 2.81E-01 |
| Glycated haemoglobin | Cancer | IVW | 1.66E-02 |
| Glycated haemoglobin | Cancer | MR Egger | 1.07E-01 |
| Glycated haemoglobin | Cancer | WM | 1.14E-01 |
| Abbreviation: MR: Mendelian randomization; SNP: single nucleotide polymorphism; IVW: inverse-variance weighted; WM: Weighted median; CAD: Coronary artery disease. | | | |

**Supplementary Table 4**. The results of Steiger directionality test

| Exposure | Outcome | snp_r^2^.  exposure | snp_r^2^.  outcome | correct_causal_direction | steiger_pval |
| --- | --- | --- | --- | --- | --- |
| Telomere length | CAD | 0.03198 | 0.00215 | TRUE | 0 |
| Sleeplessness or insomnia | CAD | 0.00347 | 0.00051 | TRUE | 2.65E-36 |
| Sitting height | CAD | 0.11652 | 0.00589 | TRUE | 0 |
| Weight | CAD | 0.06178 | 0.00552 | TRUE | 0 |
| Trunk fat-free mass | CAD | 0.09274 | 0.0058 | TRUE | 0 |
| Trunk predicted mass | CAD | 0.09235 | 0.00569 | TRUE | 0 |
| Birth weight of first child | CAD | 0.01426 | 0.00146 | TRUE | 2.51E-120 |
| Standing height | CAD | 0.19279 | 0.00773 | TRUE | 0 |
| Mean sphered cell volume | CAD | 0.10586 | 0.00577 | TRUE | 0 |
| Glycated haemoglobin | CAD | 0.0928 | 0.00507 | TRUE | 0 |
| Telomere length | Cancer | 0.03197 | 0.00137 | TRUE | 0 |
| Sleeplessness or insomnia | Cancer | 0.00347 | 0.00012 | TRUE | 2.01E-89 |
| Sitting height | Cancer | 0.11439 | 0.00232 | TRUE | 0 |
| Weight | Cancer | 0.06058 | 0.00185 | TRUE | 0 |
| Trunk fat-free mass | Cancer | 0.09024 | 0.00261 | TRUE | 0 |
| Trunk predicted mass | Cancer | 0.09023 | 0.00261 | TRUE | 0 |
| Birth weight of first child | Cancer | 0.0144 | 0.00017 | TRUE | 6.78E-264 |
| Standing height | Cancer | 0.19115 | 0.00314 | TRUE | 0 |
| Mean sphered cell volume | Cancer | 0.10487 | 0.00199 | TRUE | 0 |
| Glycated haemoglobin | Cancer | 0.08825 | 0.00149 | TRUE | 0 |

**Supplementary Table 5.** Heterogeneity test results

| Exposure | Outcome | Q_*P* |
| --- | --- | --- |
| Mean sphered cell volume | CAD | 3.86E-40 |
| Weight | CAD | 3.94E-29 |
| Glycated haemoglobin | CAD | 1.22E-23 |
| Sleeplessness / insomnia | CAD | 5.45E-04 |
| Birth weight of first child | CAD | 3.97E-14 |
| Sitting height | CAD | 8.64E-18 |
| Standing height | CAD | 1.22E-19 |
| Telomere length | CAD | 1.74E-09 |
| Trunk fat-free mass | CAD | 1.03E-22 |
| Trunk predicted mass | CAD | 1.05E-21 |
| Mean sphered cell volume | Cancer | 4.09E-32 |
| Weight | Cancer | 2.83E-20 |
| Glycated haemoglobin | Cancer | 6.13E-17 |
| Sleeplessness / insomnia | Cancer | 3.58E-01 |
| Birth weight of first child | Cancer | 1.37E-01 |
| Sitting height | Cancer | 6.97E-21 |
| Standing height | Cancer | 2.02E-27 |
| Telomere length | Cancer | 1.07E-13 |
| Trunk fat-free mass | Cancer | 2.89E-32 |
| Trunk predicted mass | Cancer | 2.88E-33 |
| Abbreviation: CAD: Coronary artery disease. | | |

**Supplementary Table** **6**. The pleiotropy of 10 potential factors

| Exposure | Outcome | Egger intercept | SE | P |
| --- | --- | --- | --- | --- |
| Mean sphered cell volume | CAD | -0.0026 | 0.0019 | 1.68E-01 |
| Weight | CAD | -0.0016 | 0.0024 | 5.13E-01 |
| Glycated haemoglobin | CAD | 0.0031 | 0.0018 | 8.15E-02 |
| Sleeplessness / insomnia | CAD | -0.0048 | 0.0100 | 6.35E-01 |
| Birth weight of first child | CAD | -0.0027 | 0.0129 | 8.36E-01 |
| Sitting height | CAD | 0.0013 | 0.0018 | 4.63E-01 |
| Standing height | CAD | -0.0013 | 0.0013 | 3.40E-01 |
| Telomere length | CAD | -0.0006 | 0.0027 | 8.26E-01 |
| Trunk fat-free mass | CAD | -0.0004 | 0.0020 | 8.27E-01 |
| Trunk predicted mass | CAD | 0.0006 | 0.0020 | 7.52E-01 |
| Mean sphered cell volume | Cancer | -0.0002 | 0.0012 | 8.50E-01 |
| Weight | Cancer | -0.0008 | 0.0015 | 5.96E-01 |
| Glycated haemoglobin | Cancer | -0.0002 | 0.0011 | 8.90E-01 |
| Sleeplessness / insomnia | Cancer | -0.0054 | 0.0039 | 1.72E-01 |
| Birth weight of first child | Cancer | 0.0042 | 0.0048 | 3.90E-01 |
| Sitting height | Cancer | 0.0009 | 0.0012 | 4.53E-01 |
| Standing height | Cancer | 0.0018 | 0.0009 | 4.94E-02 |
| Telomere length | Cancer | -0.0027 | 0.0018 | 1.44E-01 |
| Trunk fat-free mass | Cancer | 0.0006 | 0.0014 | 6.47E-01 |
| Trunk predicted mass | Cancer | 0.00004 | 0.0014 | 9.74E-01 |
| Abbreviation: SE: standard error; CAD: Coronary artery disease. | | | | |

**Supplementary Table 7.** MR-PRESSO analyses between exposures and outcomes with outlier

| Exposure | Outcome | MR Analysis | *p*-value |
| --- | --- | --- | --- |
| Mean sphered cell volume | CAD | Outlier-corrected | 1.03E-02 |
| Weight | CAD | Outlier-corrected | 1.90E-06 |
| Glycated haemoglobin | CAD | Outlier-corrected | 2.21E-11 |
| Sleeplessness / insomnia | CAD | Outlier-corrected | 8.36E-02 |
| Birth weight of first child | CAD | Outlier-corrected | 2.19E-03 |
| Sitting height | CAD | Outlier-corrected | 2.33E-09 |
| Standing height | CAD | Outlier-corrected | 2.61E-14 |
| Telomere length | CAD | Outlier-corrected | 2.40E-03 |
| Trunk fat-free mass | CAD | Outlier-corrected | 1.57E-03 |
| Trunk predicted mass | CAD | Outlier-corrected | 2.29E-03 |
| Mean sphered cell volume | Cancer | Outlier-corrected | 3.51E-02 |
| Weight | Cancer | Outlier-corrected | 1.81E-02 |
| Glycated haemoglobin | Cancer | Outlier-corrected | 2.36E-02 |
| Sitting height | Cancer | Outlier-corrected | 4.70E-05 |
| Standing height | Cancer | Outlier-corrected | 4.66E-05 |
| Telomere length | Cancer | Outlier-corrected | 4.03E-20 |
| Trunk fat-free mass | Cancer | Outlier-corrected | 2.57E-05 |
| Trunk predicted mass | Cancer | Outlier-corrected | 9.65E-05 |

**Supplementary Table 8.** MR analysis results after excluding SNPs showing associations with CAD or cancer

| Exposure | Outcome | Method | *p* |
| --- | --- | --- | --- |
| Mean sphered cell volume | CAD | IVW | 2.36E-02 |
| Mean sphered cell volume | CAD | MR Egger | 4.77E-01 |
| Mean sphered cell volume | CAD | WM | 6.09E-01 |
| Weight | CAD | IVW | 3.86E-05 |
| Weight | CAD | MR Egger | 3.29E-01 |
| Weight | CAD | WM | 7.52E-07 |
| Glycated haemoglobin | CAD | IVW | 1.09E-12 |
| Glycated haemoglobin | CAD | MR Egger | 5.11E-03 |
| Glycated haemoglobin | CAD | WM | 5.33E-03 |
| Sleeplessness / insomnia | CAD | IVW | 7.20E-02 |
| Sleeplessness / insomnia | CAD | MR Egger | 7.85E-01 |
| Sleeplessness / insomnia | CAD | WM | 3.88E-02 |
| Birth weight of first child | CAD | IVW | 7.20E-02 |
| Birth weight of first child | CAD | MR Egger | 5.18E-01 |
| Birth weight of first child | CAD | WM | 2.07E-01 |
| Sitting height | CAD | IVW | 4.83E-08 |
| Sitting height | CAD | MR Egger | 4.58E-03 |
| Sitting height | CAD | WM | 4.78E-06 |
| Standing height | CAD | IVW | 4.01E-13 |
| Standing height | CAD | MR Egger | 1.97E-02 |
| Standing height | CAD | WM | 1.22E-06 |
| telomere length | CAD | IVW | 4.13E-03 |
| telomere length | CAD | MR Egger | 8.35E-02 |
| telomere length | CAD | WM | 1.97E-01 |
| Trunk fat-free mass | CAD | IVW | 2.12E-03 |
| Trunk fat-free mass | CAD | MR Egger | 9.00E-02 |
| Trunk fat-free mass | CAD | WM | 3.21E-02 |
| Trunk predicted mass | CAD | IVW | 2.72E-03 |
| Trunk predicted mass | CAD | MR Egger | 3.86E-02 |
| Trunk predicted mass | CAD | WM | 1.49E-01 |
| Mean sphered cell volume | Cancer | IVW | 3.98E-02 |
| Mean sphered cell volume | Cancer | MR Egger | 3.18E-01 |
| Mean sphered cell volume | Cancer | WM | 9.20E-03 |
| Weight | Cancer | IVW | 1.53E-02 |
| Weight | Cancer | MR Egger | 7.17E-02 |
| Weight | Cancer | WM | 4.76E-02 |
| Glycated haemoglobin | Cancer | IVW | 2.42E-02 |
| Glycated haemoglobin | Cancer | MR Egger | 1.04E-01 |
| Glycated haemoglobin | Cancer | WM | 1.98E-01 |
| Birth weight of first child | Cancer | IVW | 4.84E-03 |
| Birth weight of first child | Cancer | MR Egger | 9.40E-01 |
| Birth weight of first child | Cancer | WM | 1.08E-02 |
| Sitting height | Cancer | IVW | 5.26E-04 |
| Sitting height | Cancer | MR Egger | 4.45E-01 |
| Sitting height | Cancer | WM | 2.57E-01 |
| Standing height | Cancer | IVW | 1.30E-04 |
| Standing height | Cancer | MR Egger | 6.94E-01 |
| Standing height | Cancer | WM | 9.64E-02 |
| telomere length | Cancer | IVW | 8.44E-16 |
| telomere length | Cancer | MR Egger | 2.40E-07 |
| Telomere length | Cancer | WM | 8.06E-13 |
| Trunk fat-free mass | Cancer | IVW | 8.15E-05 |
| Trunk fat-free mass | Cancer | MR Egger | 8.05E-01 |
| Trunk fat-free mass | Cancer | WM | 4.20E-02 |
| Trunk predicted mass | Cancer | IVW | 2.81E-04 |
| Trunk predicted mass | Cancer | MR Egger | 5.73E-01 |
| Trunk predicted mass | Cancer | WM | 5.65E-02 |
| Abbreviation: MR: Mendelian randomization; SNP: single nucleotide polymorphism; IVW: inverse-variance weighted; WM: Weighted median; CAD: Coronary artery disease. | | | |


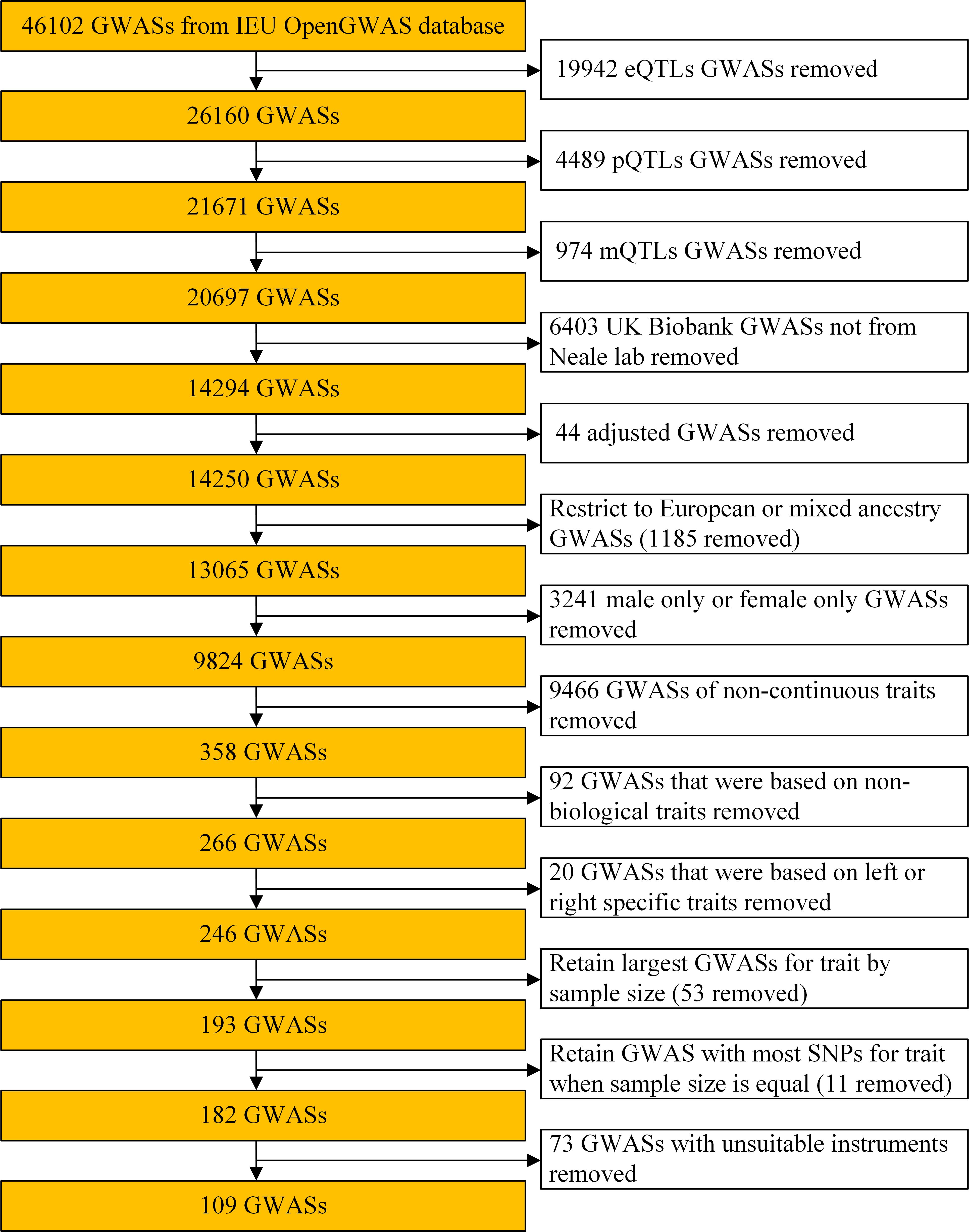


**Supplementary Figure 1.** Flowchart showing the trait selection procedure for causal analyses. GWAS: genome-wide association study.


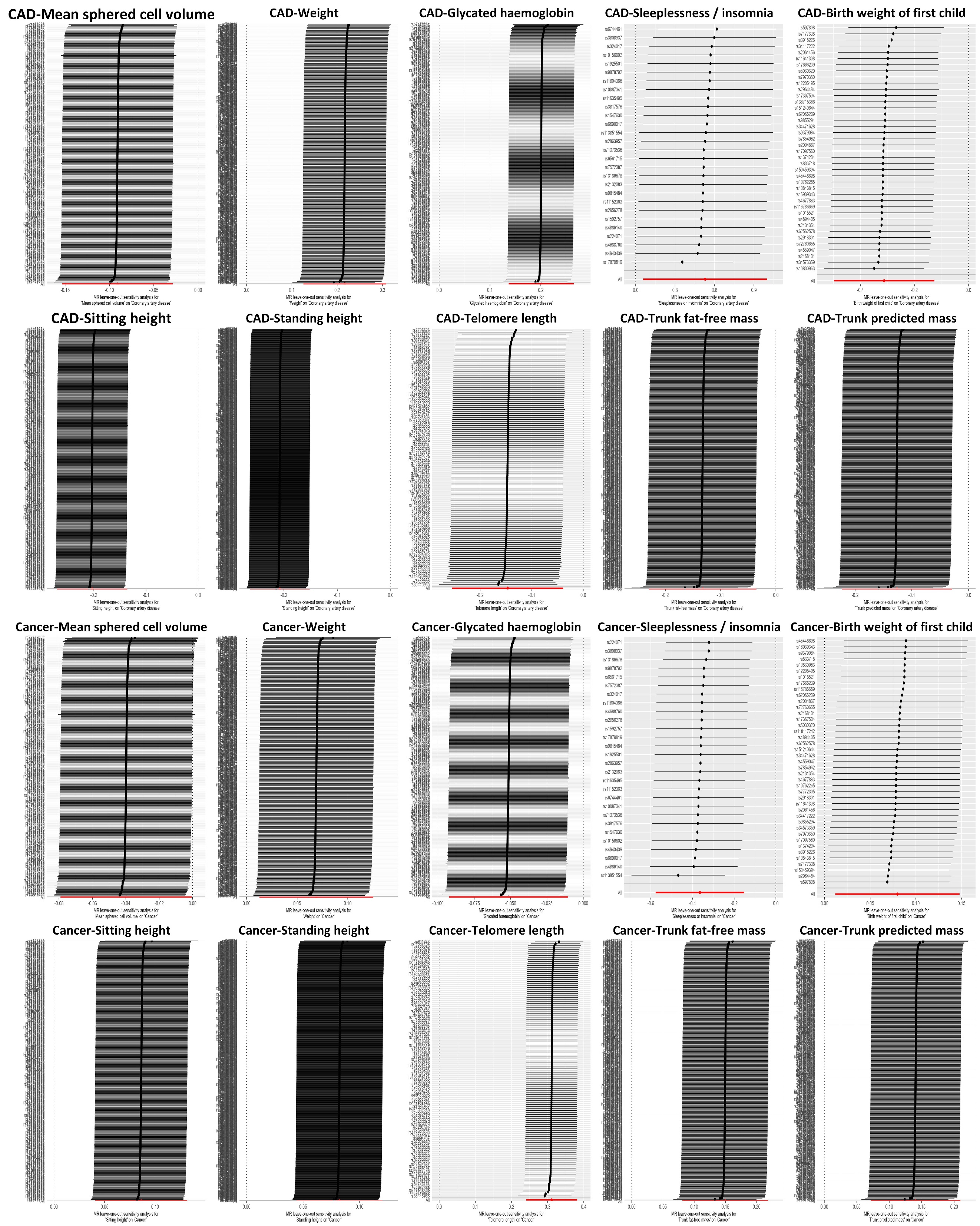


**Supplementary Figure 2.** Leave-one-out sensitivity analysis examining the causal estimates of 10 factors on both CAD and cancer by the IVW method after exclude a specific SNP from the analysis. The red line represents the IVW estimate of all SNPs on each outcome. MR: Mendelian randomization; SNP: single nucleotide polymorphism; IVW: inverse-variance weighted; CAD: coronary artery disease.
